# Association of Elevated Serum Branched-chain Amino Acid Levels With Longitudinal Skeletal Muscle Loss

**DOI:** 10.1210/jendso/bvad178

**Published:** 2024-01-11

**Authors:** Dan Imai, Naoko Nakanishi, Natsuko Shinagawa, Shinta Yamamoto, Takahiro Ichikawa, Madoka Sumi, Takaaki Matsui, Yukako Hosomi, Yuka Hasegawa, Chihiro Munekawa, Tomoki Miyoshi, Takuro Okamura, Noriyuki Kitagawa, Yoshitaka Hashimoto, Hiroshi Okada, Norihiro Sakui, Ryoichi Sasano, Masahide Hamaguchi, Michiaki Fukui

**Affiliations:** Department of Endocrinology and Metabolism, Graduate School of Medical Science, Kyoto Prefectural University of Medicine, Kyoto, 602-8566, Japan; Department of Endocrinology and Metabolism, Graduate School of Medical Science, Kyoto Prefectural University of Medicine, Kyoto, 602-8566, Japan; Department of Endocrinology and Metabolism, Graduate School of Medical Science, Kyoto Prefectural University of Medicine, Kyoto, 602-8566, Japan; Department of Endocrinology and Metabolism, Graduate School of Medical Science, Kyoto Prefectural University of Medicine, Kyoto, 602-8566, Japan; Department of Endocrinology and Metabolism, Graduate School of Medical Science, Kyoto Prefectural University of Medicine, Kyoto, 602-8566, Japan; Department of Endocrinology and Metabolism, Graduate School of Medical Science, Kyoto Prefectural University of Medicine, Kyoto, 602-8566, Japan; Department of Endocrinology and Metabolism, Graduate School of Medical Science, Kyoto Prefectural University of Medicine, Kyoto, 602-8566, Japan; Department of Endocrinology and Metabolism, Graduate School of Medical Science, Kyoto Prefectural University of Medicine, Kyoto, 602-8566, Japan; Department of Endocrinology and Metabolism, Graduate School of Medical Science, Kyoto Prefectural University of Medicine, Kyoto, 602-8566, Japan; Department of Endocrinology and Metabolism, Graduate School of Medical Science, Kyoto Prefectural University of Medicine, Kyoto, 602-8566, Japan; Department of Endocrinology and Metabolism, Graduate School of Medical Science, Kyoto Prefectural University of Medicine, Kyoto, 602-8566, Japan; Department of Diabetology and Endocrinology, Kyoto Okamoto Memorial Hospital, Kyoto, 613-0034, Japan; Department of Endocrinology and Metabolism, Graduate School of Medical Science, Kyoto Prefectural University of Medicine, Kyoto, 602-8566, Japan; Department of Endocrinology and Metabolism, Graduate School of Medical Science, Kyoto Prefectural University of Medicine, Kyoto, 602-8566, Japan; Department of Diabetology, Kameoka Municipal Hospital, Kyoto, 621-8585, Japan; Department of Endocrinology and Metabolism, Graduate School of Medical Science, Kyoto Prefectural University of Medicine, Kyoto, 602-8566, Japan; Department of Diabetes and Endocrinology, Matsushita Memorial Hospital, Moriguchi, 570-8540, Japan; Department of Endocrinology and Metabolism, Graduate School of Medical Science, Kyoto Prefectural University of Medicine, Kyoto, 602-8566, Japan; AiSTI SCIENCE Co., Ltd, Wakayama, 640-8390, Japan; AiSTI SCIENCE Co., Ltd, Wakayama, 640-8390, Japan; Department of Endocrinology and Metabolism, Graduate School of Medical Science, Kyoto Prefectural University of Medicine, Kyoto, 602-8566, Japan; Department of Endocrinology and Metabolism, Graduate School of Medical Science, Kyoto Prefectural University of Medicine, Kyoto, 602-8566, Japan

**Keywords:** branched-chain amino acids, skeletal muscle mass, body mass index, skeletal muscle index, insulin resistance

## Abstract

**Context:**

Branched-chain amino acids (BCAA) are substrates for protein synthesis. Although their intake may contribute to an increase in skeletal muscle mass, elevated serum BCAA levels have been reported to be associated with insulin resistance, potentially resulting in decreased skeletal muscle mass.

**Objective:**

This study aimed to explore the association between elevated serum BCAA levels and longitudinal skeletal muscle loss.

**Design and Setting:**

A cohort analysis was conducted, in which serum amino acids were analyzed in healthy individuals who underwent a medical health checkup at Kameoka Municipal Hospital (HOZUGAWA study), Japan.

**Patients:**

Seventy-one participants (37 men and 34 women) underwent follow-up checkups after the baseline visit. The follow-up duration was 1.2 ± .4 years.

**Main Outcome Measures:**

The relationship between fasting baseline serum BCAA levels and lifestyle factors, body composition, blood test results, dietary history, and changes in skeletal muscle mass was evaluated.

**Results:**

In both men and women, serum BCAA levels were positively correlated with body weight, body mass index, skeletal muscle mass index (SMI), and serum triglycerides but inversely correlated with serum high-density lipoprotein cholesterol. In men, fasting serum BCAA levels were inversely associated with the rate of change in SMI (adjusted β = −.529, *P* = .006), and elevated BCAA levels were independently associated with a longitudinal decrease in skeletal muscle mass (odds ratio: 1.740; 95% confidence interval: 1.023-2.960 per 50 nmol/mL serum BCAAs increase).

**Conclusion:**

Increased circulating BCAAs could be an indicator of skeletal muscle loss in men.

In geriatric research and clinical settings, skeletal muscle loss is a major public health issue among older adults and associated with poor quality of life [1]. Effective ways to maintain skeletal muscle need to be established.

Branched-chain amino acids (BCAA; valine, leucine, and isoleucine) are substrates for protein synthesis; thus, their intake promotes synthesis of skeletal muscle proteins. For instance, supplementation with essential amino acids, including BCAA, increases lean body mass and basal muscle protein synthesis in old individuals [2]. Furthermore, the intake of energy and BCAAs is positively correlated with skeletal muscle index (SMI) [3]. Protein supplementation increases muscle mass and strength during prolonged resistance training in both young and old individuals [4]. Activation of the mammalian target of rapamycin complex 1 by BCAA plays a fundamental role in stimulating human skeletal muscle protein synthesis in response to essential amino acids ingestion [5]. Supplementation of protein/amino acids with resistance exercise exhibits clear protein dose-dependent effects on the translational regulation of protein synthesis via the mammalian target of rapamycin complex 1 pathway, which is crucial for muscle growth [6].

In contrast, several observational studies have shown that serum BCAA concentrations are high during conditions of insulin resistance, such as obesity and glucose intolerance [7-9]. Furthermore, circulating levels of BCAAs are associated with poor metabolic health and future insulin resistance or type 2 diabetes mellitus [10-12]. Insulin resistance elevates serum BCAA levels that induce impaired glucose tolerance [13], thus forming a causal loop. Lowering BCAA and branched-chain ketoacid levels by feeding a BCAA-restricted diet to rodent models of obesity have apparent beneficial effects on glucose levels [14]. Despite knowing the effects of elevated serum BCAA levels, their cause remains unidentified. BCAA homeostasis and plasma levels are determined based on their appearance and disappearance, which are affected by several processes. Factors contributing to the appearance in the blood include food intake, gut microbial synthesis, and tissue protein breakdown, whereas processes regulating the disappearance are protein synthesis from the amino acid, BCAA excretion, and catabolism [15, 16]. In an insulin-resistant state, muscle protein synthesis is significantly reduced and the ability of insulin to decrease muscle protein breakdown is impaired [17].

Thus, a paradox regarding the regulation of BCAA and muscle mass exists. Although their intake may contribute to an increase in skeletal muscle mass, elevated serum BCAA levels have been reported to be associated with insulin resistance, potentially resulting in decreased skeletal muscle mass.

This study aimed to evaluate the relationship between serum BCAA levels and changes in skeletal muscle mass in healthy individuals. We examined the relationship between fasting serum BCAA concentrations and obesity, glucose intolerance, and oral protein intake and investigated whether circulating BCAAs are an indicator of longitudinal skeletal muscle mass loss.

## Methods

### Study Design

We performed a cohort analysis with participants who underwent a medical health checkup at Kameoka Municipal Hospital, Japan. After informed consent was obtained from the participants and their personally identifiable information was removed, the checkup results were saved in a database, and the sera collected from each participant in the overnight-fasting state were stored at −80 °C. We named this longitudinal cohort analysis the HOZUGAWA study. This study was approved by the ethics committee of the Kyoto Prefectural University of Medicine (approval no. ERB-C-1503) and was performed as per the Declaration of Helsinki. Serum amino acid analysis using gas chromatography–mass spectrometry (GC/MS) was performed on 196 participants who underwent a medical health checkup between September 2019 and February 2021. The baseline visit was defined as the checkup date when serum amino acid levels were examined. Of the 196 participants, 72 underwent follow-up checkups after the baseline visit, with a follow-up period of 6 months or longer. One participant with high blood sugar (>250 mg/dL) was excluded to eliminate individuals with acute metabolic imbalance. Finally, between serum BCAA levels and changes in skeletal muscle mass were investigated in 71 individuals.

### Data Collection

We examined age, sex, family history of diabetes, lifestyle factors (smoking and regular exercise), waist circumference, and blood pressure. Blood samples were collected from participants in the overnight-fasting state, and aspartate aminotransferase (AST), alanine aminotransferase (ALT), γ-glutamyl transferase, fasting blood glucose, hemoglobin A1c (HbA1c), creatinine, total cholesterol, triglycerides, and high-density lipoprotein (HDL) cholesterol were measured. The estimated glomerular filtration rate (eGFR) was calculated using the Japanese Society of Nephrology equation, as follows: eGFR = 194 × serum creatinine^−1.094^× age^−.287^ × .739 (for women) (mL/min/1.73 m^2^). The participants were asked about their exercise habits, and those who exercised regularly 2 or more times a week were categorized in the regularly exercising group. Body weight (BW), appendicular muscle mass, and body fat mass were measured using a multifrequency impedance body composition analyzer (InBody 720, InBody Japan, Tokyo, Japan). Multifrequency impedance body composition analyzer corroborated the results of the dual-energy X-ray absorptiometry and hence was validated [18]. Body mass index (BMI) was defined as body weight (kg) ÷ height-squared (m^2^) and SMI as appendicular muscle mass (kg) ÷ height-squared (m^2^). Ideal body weight (IBW) was defined as 22× patient height squared (m^2^) [19]. The change in SMI (kg/m^2^ per year) was calculated as (follow-up SMI [kg/m^2^] − baseline SMI [kg/m^2^]) divided by the follow-up period (years). The rate of change in SMI (%) was calculated as follows: (change in SMI/baseline SMI [kg/m^2^]) × 100. In addition, muscle mass loss was defined as the rate of SMI change ≥.5% [20-22].

A brief self-administered diet history questionnaire (BDHQ) was used to evaluate habitual food and nutrient intake [23]. The BDHQ was used to estimate the dietary intake and variations in the consumption of 58 food items during the month prior to participation using an algorithm based on the Standard Tables of Food Composition 2010 in Japan [Standard Tables of Food Composition in Japan. Ministry of Education, Culture, Sports, Science and Technology, Tokyo, Japan (2010)]. The BDHQ has been previously validated [24]. Carbohydrate (g/day), total protein (g/day), animal protein (g/day), vegetable protein (g/day), fat (g/day), dietary fiber (g/day), and alcohol consumption (g/day) were also calculated. The energy from carbohydrates, proteins, and fats was calculated as carbohydrate (g/day) × 4, protein (g/day) × 4, and fat intake (g/day) × 9. Each intake/total energy intake (%) was calculated as follows: intake (kcal/day) divided by total energy intake (kcal/day) × 100. Energy intake (kcal/IBW/day) and (kcal/actual BW/day) were defined as energy intake (kcal/day) divided by IBW (kg) and BW (kg), respectively. Alcohol consumption was evaluated according to the amount and type of alcohol consumed per week during the month prior to participation, and the mean ethanol intake per week was estimated.

### Determining BCAA Serum Levels

Serum amino acids, including BCAA, were analyzed using GC/MS on the Agilent 7890B/7000D System (Agilent Technologies, Santa Clara, CA, USA). Serum samples (50 μL) were added to 500 μL of acetonitrile and 500 μL of distilled water. Then, the samples were shaken at 1000 rpm for 30 minutes at 37 °C and centrifuged at 14 000 rpm for 3 minutes at 20 to 25 °C. The supernatant (500 uL) was separated, added to 500 μL of acetonitrile, and shaken at 1000 rpm for 3 minutes at 37 °C. The samples were then centrifuged at room temperature (approximately 22 °C) for 3 minutes at 14 000 rpm, and the pH was adjusted to 8 using .1 mol/L NaOH.

The amino acid concentrations were then determined using GC/MS with online solid-phase extraction (SPE) method. In the SPE–GC system SPL-M100 (AiSTI SCIENCE, Wakayama, Japan), SPE and injection into the GC/MS system were automatically performed after the sample was added to the vial and placed on an autosampler tray. Flash-SPE ACXs (AiSTI SCIENCE) were used for the solid stratification, and 50 µL aliquots of each of the aforementioned sample extracts were loaded onto the solid phase and washed with acetonitrile and water (1:1). Then, the samples were dehydrated with acetonitrile and impregnated with 4 μL of .5% methoxyamine–pyridine solution. Then, N-methyl-N-trimethylsilyltrifluoroacetamide and toluene (3:1) were added to the solid phase to perform methoxylation and trimethylsilylation during derivatization and eluted with hexane. The final product was injected through a PTV injector, LVI-S250 (AiSTI SCIENCE), and the temperature was maintained at 220 °C for .5 minutes, gradually increased to 290 °C at 50 °C/min, and maintained at 220 °C 16 minutes. The samples were then loaded onto a capillary column, Vf-5 ms [30 m × .25 mm (inner diameter) × .25 μm (membrane thickness); Agilent Technologies]. The column temperature was maintained at 80 °C for 3 minutes and then increased gradually by 25 °C/min to 190 °C, by 3 °C/min to 220 °C, and by 15 °C/min to 310 °C, which was then maintained for 4.6 minutes. The sample was injected in the split mode at a split ratio of 50:1. Each amino acid was detected in the scan mode (m/z 70-470). All results were normalized to the peak height of norleucine (.01 mM for each amino acid).

### Statistical Analyses

Continuous values are presented as mean ± SD, whereas categorical values are presented as n (%). Student's *t*-tests were used to analyze statistical differences in continuous variables. The correlation coefficients between fasting serum BCAA concentrations and the aforementioned factors were calculated using Pearson correlation analysis if values showed a normal distribution or, alternatively, using Spearman correlation analysis. We used unadjusted and adjusted linear regression analyses to assess variables associated with changes in SMI between the baseline and second visits in men and women, respectively. Logistic regression analysis was performed to calculate unadjusted and adjusted odds ratios and 95% confidence intervals (CIs) for the effect of fasting serum BCAA concentrations on the muscle mass loss, adjusting for age, exercise, fasting blood glucose, SMI at baseline, protein intake, and total energy intake as confounders. We selected covariates, using the knowledge-based outcome approach. We also excluded covariates that exhibited strong multicollinearity. All statistical analyses were performed using the Statistical Package for the Social Sciences (SPSS) version 28.0 software (IBM Corp., Armonk, NY, USA).

## Results

A total of 71 participants who underwent a medical health checkup at the Kameoka Municipal Hospital between September 2019 and February 2021 were enrolled in this study. The follow-up duration was 1.2 ± .4 years. Of 71 participants, 37 (52.1%) participants were male and 34 (47.9%) were female. The baseline characteristics of the study participants are shown in Table 1. The average age was 64.6 ± 10.9 years for men and 61.1 ± 9.4 years for women, and the average BMI was 24.1 ± 3.5 for men and 21.9 ± 3.9 for women. The dietary habits of the participants are presented in Table 2. The mean total energy intake was 32.0 ± 9.6 kcal/IBW/day for men and 29.3 ± 7.0 kcal/IBW/day for women. and the mean protein intake was 4.7 ± 1.7 kcal/IBW/day for men and 5.0 ± 1.8 kcal/IBW/day for women.

**Table 1.** Clinical characteristics of the study participants

Characteristic	All participants (n = 71)	Men (n = 37)	Women (n = 34)
Age (years)	62.9 (10.3)	64.6 (10.9)	61.1 (9.4)
Habit of smoking, current smoker, n (%)	9 (12.7)	8 (21.6)	1 (3.0)
Habit of exercise, yes, n (%)	25 (35.2)	14 (37.8)	11 (32.4)
History of diabetes, n (%)	6 (8)	5 (13)	1 (3)
Waist circumference (cm)	85.2 (10.8)	88.8 (10.2)	81.4 (10.3)
Systolic blood pressure (mmHg)	132.0 (17.5)	137.0 (15.8)	126.1 (15.7)
Diastolic blood pressure (mmHg)	81.5 (11.9)	86.6 (11.2)	74.7 (9.3)
Fasting blood glucose (mg/dL)	100 (94.0-109.0)	104 (97-114)	96.5 (91.0-102.3)
HbA1c (%)	5.7 (5.6-6.0)	5.9 (5.6-6.1)	5.7 (5.5-6.0)
HbA1c (mmol/mol)	38.0 (37.0-42.0)	40.0 (37.0-43.0)	38.0 (36.0-42.0)
AST (mg/dL)	22.0 (17.7-25.0)	22.5 (18.2-28.8)	22.0 (17.0-24.0)
ALT (mg/dL)	19.0 (15.0-23.2)	20.5 (15.5-28.8)	17.0 (14.8-20.0)
γ-GTP (mg/dL)	24.0 (18.0-40.0)	27.0 (22.0-48.5)	20.0 (14.8-25.8)
Creatinine	.8 (.2)	.9 (.2)	.7 (.1)
eGFR (mL/min/1.73 m^2^)	63.7 (15.9)	61.0 (19.2)	66.6 (10.9)
Total cholesterol (mg/dL)	212.6 (32.6)	212.0 (37.2)	213.2 (26.2)
Triglycerides (mg/dL)	83.0 (66.0-125.0)	109.0 (72.5-156.5)	71.5 (60.3-98.8)
HDL cholesterol (mg/dL)	70.3 (20.3)	66.0 (22.0)	74.7 (17.7)
Body composition			
Body weight (kg)	61.3 (13.7)	68.3 (12.6)	53.8 (10.6)
Body mass index (kg/m^2^)	23.0 (3.8)	24.1 (3.5)	21.9 (3.9)
Body fat mass (kg)	16.9 (6.9)	17.2 (6.5)	16.7 (7.5)
Percentage of body fat mass	27.2 (7.2)	24.6 (5.5)	30.0 (7.9)
Lean body mass (kg)	42.0 (9.0)	48.3 (7.3)	35.0 (4.3)
Fat-free mass (kg)	44.5 (9.5)	51.0 (7.8)	37.1 (4.6)
Skeletal muscle mass (kg)	24.3 (5.8)	28.4 (4.7)	19.8 (2.7)
SMI (kg/m^2^)	6.8 (1.1)	7.6 (.9)	5.9 (.7)
Percentage of skeletal muscle mass (%)	29.3 (3.5)	31.8 (2.6)	27.1 (3.1)

Abbreviations: ALT, alanine aminotransferase; AST, aminotransferase; eGFR, estimated glomerular filtration rate; γ-GTP, γ-glutamyl transferase; HbA1c, hemoglobin A1c; HDL, high-density lipoprotein; SMI, skeletal muscle mass index.

Data are presented as n (%), mean ± SD, or median (25th, 75th).

**Table 2.** Habitual diet of the study participants

Habitual diet intake	All participants (n = 71)	Men (n = 37)	Women (n = 34)
Total energy intake (kcal/day)	1782.3 (493.0)	1968.3 (524.5)	1580.0 (366.5)
Total energy intake (kcal/IBW/day)	30.7 (8.5)	32.0 (9.6)	29.3 (7.0)
Total energy intake (kcal/actual BW/day)	30.2 (9.9)	29.9 (10.2)	30.5 (9.7)
Carbohydrate intake (g/day)	232.1 (80.5)	255.7 (96.6)	206.3 (47.6)
Carbohydrate intake/Total energy intake (%)	52.0 (8.8)	51.5 (10.7)	52.6 (6.2)
Carbohydrate intake (kcal/IBW/day)	16.0 (5.2)	16.6 (6.5)	15.3 (3.3)
Carbohydrate intake (kcal/actual BW/day)	15.6 (5.6)	15.5 (6.5)	15.8 (4.7)
Protein intake (g/day)	69.4 (22.6)	72.1 (23.0)	66.5 (22.1)
Protein intake/Total energy intake (%)	15.6 (2.9)	14.7(2.8)	16.6 (4.7)
Protein intake (kcal/IBW/day)	4.8 (1.7)	4.7 (1.7)	5.0 (1.8)
Protein intake (kcal/actual BW/day)	4.8 (2.1)	4.4 (1.9)	5.2 (2.3)
Animal protein intake (g/day)	40.9 (17.6)	41.1 (17.5)	40.6 (17.9)
Vegetable protein intake (g/day)	28.5 (8.5)	31.0 (9.2)	25.9 (7.0)
Fat intake (g/day)	52.9 (15.6)	55.0 (15.1)	50.6 (16.0)
Fat intake/total energy intake (%)	27.0 (4.9)	25.6 (5.0)	28.5 (4.4)
Fat intake (kcal/IBW/day)	8.2 (2.7)	8.1 (2.5)	8.4 (2.9)
Fat intake (kcal/actual BW/day)	8.1 (3.2)	7.5 (2.6)	8.8 (3.6)
Dietary fiber intake (g/day)	11.5 (4.4)	11.9 (4.9)	11.1 (3.8)
Alcohol consumption (g/day)	.0 (.0-16.7)	4.5 (.0-38.6)	.0 (.0-.3)

Abbreviations: BW, body weight; IBW, ideal body weight.

Data are presented as mean ± SD or median (25th, 75th).

The fasting serum BCAA levels of the study participants are listed in Table 3. The mean BCAA level was 384.1 ± 113.7 µmol/L. Valine, leucine, and isoleucine concentrations were higher in males than in females (Table 3). The BCAA level in men (425.1 ± 17.4 µmol/L) was significantly higher than that in women (339.4 ± 89.1 µmol/L) (*P* = .001). The significant difference in BCAA levels between men and women led us to investigate the impact of serum BCAAs separately in men and women.

**Table 3.** Fasting serum BCAA levels of the study participants

Amino acid	All participants (n = 71)	Men (n = 37)	Women (n = 34)	*P*
Valine (μmol/L)	215.3 (63.0)	236.0 (66.8)	192.7 (50.5)	.003
Leucine (μmol/L)	115.4 (34.5)	128.4 (36.1)	101.3 (26.7)	.001
Isoleucine (μmol/L)	53.4 (17.8)	60.7 (2.7)	45.5 (2.8)	<.001
BCAA (μmol/L)	384.1 (113.7)	425.1 (17.4)	339.4 (89.1)	.001

Abbreviations: BCAA, branched-chain amino acids.

Data are presented as mean (SD). *P-*values were calculated using Student's *t*-test.

The correlations between fasting serum BCAA levels and the baseline characteristics of the participants are summarized in Table 4. In men, they were positively correlated with waist circumference (r = .373, *P* = .023), body weight (r = .414, *P* = .011), BMI (r = .333, *P* = .044), SMI (r = .480, *P* = .003) and serum triglycerides (r = .403, *P* = .013) and negatively correlated with serum HDL-cholesterol (r = −.504, *P* = .001). In women, they were positively correlated with body weight (r = .409, *P* = .016), BMI (r = .418, *P* = .014), SMI (r = .437, *P* = .010) serum γ-GTP (r = .397, *P* = .020) and serum triglycerides (r = .477, *P* = .004) and negatively correlated with serum HDL-cholesterol (r = −.469, *P* = .005). Notably, in women, fasting serum BCAAs were negatively correlated with total energy intake (kcal/actual BW/day) (r = −.342, *P* = .048), protein intake (kcal/actual BW/day) (r = −.362, *P* = .035), and fat intake (kcal/actual BW/day) (r = −.373, *P* = .030) (Table 5).

**Table 4.** Correlation between serum BCAA level and characteristics of the participants at baseline

Characteristic	All participants		Men		Women	
	r*^a^*	*P*	r	*P*	r	*P*
Age (years)	−.090	.456	−.238	.156	−.05	.769
Waist circumference (cm)	.428	<.001	.373	.023	.306	.079
Systolic blood pressure (mmHg)	.267	.025	.105	.536	.336	.052
Diastolic blood pressure (mmHg)	.388	<.001	.274	.100	.269	.124
AST (mg/dL)	−.096	.428	−.298	.077	.004	.983
ALT (mg/dL)	.254	.034	.189	.269	.237	.176
γ-GTP (mg/dL)	.281	.018	−.016	.927	.397	.020
Creatinine (mg/dL)	.026	.832	−.067	.693	.237	.178
eGFR (mL/min/1.73m^2^)	−.116	.336	−.017	.921	−.147	.406
Total cholesterol (mg/dL)	−.003	.979	−.020	.905	.067	.750
Triglycerides (mg/dL)	.521	<.001	.403	.013	.477	.004
Fasting blood glucose (mg/dL)	.256	.031	.161	.342	.011	.949
HbA1c (%)	.245	.039	.202	.231	.129	.465
HDL cholesterol (mg/dL)	−.492	<.001	−.504	.001	−.469	.005
Body composition						
Body weight (kg)	.525	<.001	.414	.011	.409	.016
Body mass index (kg/m^2^)	.431	<.001	.333	.044	.418	.014
Percentage of body fat mass (%)	.006	.966	.072	.671	.300	.086
Percentage of skeletal muscle mass (%)	.162	.177	.003	.984	−.17	.335
SMI (kg/m^2^)	.571	<.001	.480	.003	.437	.010

Abbreviations: ALT, alanine aminotransferase; BCAA, branched-chain amino acids; AST, aminotransferase; eGFR, estimated glomerular filtration rate; γ-GTP, γ-glutamyl transferase; HbA1c, hemoglobin A1c; SMI, skeletal muscle mass index.

^*a*^r is the correlation coefficient. *P* values were calculated using Pearson or Spearman correlation analyses.

**Table 5.** Correlation between serum BCAA level and habitual diet at baseline

Habitual diet	All participants		Men		Women	
	r*^a^*	*P*	r	*P*	r	*P*
Total energy intake (kcal/day)	.197	.100	.149	.379	−.142	.422
Total energy intake (kcal/IBW/day)	.029	.812	.049	.775	−.203	.249
Total energy intake (kcal/actual BW/day)	−.174	.146	−.071	.678	−.342	.048
Carbohydrate intake (g/day)	.300	.011	.266	.111	.048	.788
Carbohydrate intake/total energy intake (%)	.220	.066	.241	.152	.326	.060
Carbohydrate intake (kcal/IBW/day)	.177	.140	.190	.261	.004	.984
Carbohydrate intake (kcal/actual BW/day)	−.021	.863	.081	.633	−.192	.278
Protein intake (g/day)	−.049	.687	−.021	.901	−.234	.183
Protein intake/total energy intake (%)	−.308	.009	−.209	.215	−.206	.243
Protein intake (kcal/IBW/day)	−.172	.153	−.081	.636	−.272	.120
Protein intake (kcal/actual BW/day)	−.291	.014	−.159	.347	−.362	.035
Animal protein intake (g/day)	−.119	.325	−.025	.883	−.294	.091
Vegetable protein intake (g/day)	.115	.339	−.005	.975	.016	.930
Fat intake (g/day)	.037	.760	.117	.492	−.207	.241
Fat intake/total energy intake (%)	−.174	.146	−.001	.997	−.180	.308
Fat intake (kcal/IBW/day)	−.115	.340	.026	.879	−.255	.145
Fat intake (kcal/actual BW/day)	−.274	.021	−.090	.595	−.373	.030
Dietary fiber intake (g/day)	.034	.777	−.008	.963	.020	.910
Alcohol consumption (g/day)	−.050	.676	−.277	.097	−.112	.528

Abbreviations: BCAA, branched-chain amino acids; BW, body weight; IBW, ideal body weight.

^*a*^r is the correlation coefficient. *P-*values were calculated using Pearson or Spearman correlation analyses.

Regression analyses for the rate of change in SMI per year in men and women are shown in Tables 6 and 7. The follow-up duration was 1.2 ± .4 years in men and 1.3 ± .5 years in women. In men, serum BCAA levels and SMI change rate per year were negatively associated in both univariate and multivariate models [β=−.428, *P* = .008 (unadjusted) and β=−.529, *P* = .006 (adjusted)], which showed that the increase in serum BCAA levels was independently associated with a decrease in SMI. In contrast, in women, there was not a significant correlation between serum BCAA level and SMI change rate per year. The odds ratio of skeletal muscle loss with a reduction in SMI of more than .5% per year in men is shown in Table 8. Elevated serum BCAA levels were associated with skeletal muscle loss [odds ratio: 1.409; 95% confidence interval: 1.017-1.950 (unadjusted) and odds ratio: 1.740; 95% confidence interval: 1.023-2.960 (adjusted) per 50 nmol/mL serum BCAAs increase] in men.

**Table 6.** Regression analyses for the rate of change in SMI per year in men

Variable	Unadjusted Models		Adjusted Models	
	β	*P*	β	*P*
Age	−.018	.918	−.006	.975
Habit of exercise (yes)	−.178	.292	−.204	.237
Fasting blood glucose	.192	.255	−.094	.580
SMI at baseline	−.292	.080	−.112	.595
Protein intake (kcal/IBW/day)	−.019	.912	−.521	.114
Energy intake (kcal/IBW/day)	.075	.660	.585	.060
Serum BCAA level	−.428	.008	−.529	.006

Abbreviations: BCAA, branched-chain amino acids; IBW, ideal body weight; SMI, skeletal muscle mass index.

β represents linear regression coefficients. *P*-values were calculated using unadjusted and adjusted linear regression analyses.

**Table 7.** Regression analyses for the rate of change in SMI per year in women

Variables	Unadjusted models		Adjusted models	
	β	*P*	β	*P*
Age	.270	.123	−.278	.184
Habit of exercise (yes)	−.082	.646	−.119	.545
Fasting blood glucose	.048	.787	.155	.499
SMI at baseline	−.020	.909	−.147	.554
Protein intake (kcal/IBW/day)	−.089	.615	−.154	.756
Energy intake (kcal/IBW/day)	−.072	.685	.118	.799
Serum BCAA level	−.009	.959	−.028	.898

Abbreviations: BCAA, branched-chain amino acids; IBW, ideal body weight; SMI, skeletal muscle mass index.

β represents linear regression coefficients. *P-*values were calculated using unadjusted and adjusted linear regression analyses.

**Table 8.** Odds ratio for more than 0.5% rate of SMI change per year in men

	Unadjusted		Adjusted	
	OR (95% CI)	*P*	OR (95% CI)	*P*
Age (per 1 year increase)	1.028 (.966-1.094)	.379	1.074 (.953-1.210)	.240
Habit of exercise (yes)	.818 (.215-3.118)	.769	1.280 (.180-9.105)	.805
Fasting blood glucose (per 1 mg/dL increase)	.671 (.971-1.046)	.671	.995 (.937-1.057)	.875
SMI at baseline (per 1 kg/m^2^ increase)	1.646 (.732-3.702)	.228	2.666 (.481-14.773)	.262
Protein intake (per 1 kcal/IBW/day increase)	1.060 (.233-4.819)	.940	16.83 (.174-1623.0)	.226
Energy intake (per 1kcal/IBW/day increase)	.986 (.921-1.056)	.689	.854 (.639-1.039)	.132
Serum BCAA level (per 50 nmol/mL increase)	1.409 (1.017-1.950)	.039	1.704 (1.023-2.960)	.025

Abbreviations: BCAA, branched-chain amino acids; CI, confidence interval; IBW, ideal body weight; OR, odds ratio; SMI, skeletal muscle mass index.

Unadjusted and adjusted odds ratios and *P-*values were calculated using univariate and multivariate logistic regressions, respectively.

## Discussion

This study evaluated the relationship between elevated serum amino acid levels and changes in skeletal muscle mass in healthy individuals. Our results indicated an inverse correlation between the rate of change in SMI and serum BCAA levels in men, and logistic regression analysis showed that increased serum BCAA levels were independently associated with incident muscle mass loss.

As previously reported, BCAA levels are positively correlated with waist circumference, body weight, and BMI [7]. Plasma accumulation of valine, leucine, and isoleucine is associated with obesity [25] and hepatic and muscle insulin resistance [26]. Several studies have shown that elevated serum BCAA levels are associated with insulin resistance [7-9].

BCAA circulates in the blood after consuming a protein-rich meal. Lee et al reported that BCAA intake causes an increase in plasma BCAA levels in the postprandial state in mice [27]. However, to the best of our knowledge, to date, no study has investigated the significance of fasting plasma BCAA levels, particularly with respect to the amount of BCAA intake. An important finding of this study was that fasting serum BCAA levels were not correlated with protein intake in men and were rather negatively correlated with protein intake (kcal/actual BW/day) in women. These findings suggest that, in the fasting state, an increase in serum BCAA levels does not reflect a large amount of BCAA intake.

BCAA homeostasis and plasma levels are defined by BCAA appearance (BCAA intake, gut microbial synthesis, and protein breakdown) and disappearance (protein synthesis from amino acids, BCAA excretion, and catabolism) [15, 16]. A possible mechanism that could explain the relationship between elevated serum BCAA levels and decreased skeletal muscle mass is an impaired balance between protein synthesis and breakdown.

Insulin is one of the most important regulators of protein metabolism [17], and it has a specific effect of attenuating the breakdown of proteins [28, 29]. In an insulin-resistant state, muscle protein synthesis is significantly reduced, and the ability of insulin to decrease muscle protein breakdown is impaired [17]. Protein degradation is frequently increased in fasting individuals with obesity and insulin resistance and those with poorly controlled type 2 diabetes [30-32]. Taken together with our results showing a positive correlation of BCAA levels with waist circumference and serum triglycerides but a negative correlation with serum HDL-cholesterol, we suggest that an insulin-resistant state is one of the mechanisms that reduces muscle protein synthesis and induces breakdown, resulting in elevated serum BCAA levels and loss of skeletal muscle.

Other than protein turnover, a possible mechanism for elevated BCAA concentrations is dysfunctional BCAA catabolism. Animal and human studies also prove that dysfunctional BCAA catabolism in several tissues can explain the high plasma BCAA levels observed in individuals with obesity and type 2 diabetes [16]. The activity of branched-chain ketoacid dehydrogenase, which contributes to BCAA catabolism, is reduced in the liver and adipose tissue of mouse or rat models of obesity and type 2 diabetes [33-35]. A previous study using tandem mass spectrometry identified differences in protein abundance per mitochondrial mass in case of insulin resistance, including a lower abundance of complex I subunits and enzymes involved in BCAA oxidation [36].

This study observed an association between elevated BCAA levels and skeletal muscle loss in men but not in women. In women, BCAA concentrations were associated with baseline waist circumference; however, this correlation was weaker than that in men. It is also possible that the low skeletal muscle mass at baseline in women affected the results of this study.

The strength of this study is that we investigated the impact of serum BCAA concentrations on multifaceted aspects, including dietary history and body composition. Furthermore, it is valuable to assess the relationship between serum BCAA and longitudinal changes in skeletal muscle mass instead of a cross-sectional assessment in terms of providing new insight into the role of circulating BCAAs as an indicator of skeletal muscle loss. This study has some limitations. First, a small sample size and short follow-up duration were used in this study. However, it is worthwhile to comprehensively examine the significance of fasting serum amino acid levels by evaluating multiple aspects, including serum amino acid analysis using gas chromatography, dietary history, and determining longitudinal changes in SMI. Second, we did not directly assess insulin resistance. However, we consider that high waist circumference, elevated serum triglycerides, and low serum HDL-cholesterol reflect insulin resistance, which is one of the crucial mechanisms related to elevated serum BCAA levels and skeletal muscle loss.

## Conclusions

In men, but not in women, fasting serum BCAA levels were inversely associated with the rate of change in SMI, and elevated BCAA levels were independently associated with a longitudinal decrease in skeletal muscle mass. Our findings suggest that increased circulating BCAAs could be an indicator of skeletal muscle loss in men.

## Data Availability

Some or all datasets generated during and/or analyzed during the current study are not publicly available but are available from the corresponding author on reasonable request.
